# How Does the Number of Small Goals Affect National-Level Female Soccer Players in Game-Based Situations? Effects on Technical–Tactical, Physical, and Physiological Variables

**DOI:** 10.3390/s25134035

**Published:** 2025-06-28

**Authors:** Dovydas Alaune, Audrius Snieckus, Bruno Travassos, Paweł Chmura, David Pizarro, Diogo Coutinho

**Affiliations:** 1 Institute of Sport Science and Innovations, Lithuanian Sports University, 44221 Kaunas, Lithuania; dovydas.alaune@gmail.com (D.A.); a.snieckus@gmail.com (A.S.); 2 Research Center in Sports Sciences, Health Sciences and Human Development (CIDESD), 5000-801 Vila Real, Portugal; 3 Portugal Football School, Portuguese Football Federation, 4711-852 Oeiras, Portugal; 4 Department of Sports Sciences, University of Beira Interior, 6201-001 Covilhã, Portugal; 5 Department of Individual and Team Sports, Wroclaw University of Health and Sport Sciences, 51-612 Wrocław, Poland; pawel.chmura@awf.wroc.pl; 6 Department of Education and Humanities, Faculty of Social Sciences and Communication, Universidad Europea de Madrid, 28670 Madrid, Spain; davidpizarromateo@gmail.com; 7 Faculty of Education, Camilo José Cela University, 28692 Madrid, Spain; 8 Don Bosco Center for Higher Studies (CES Don Bosco), Complutense University of Madrid (UCM), 28040 Madrid, Spain; 9 Faculty of Life Sciences and Nature, University of Nebrija, 28015 Madrid, Spain; 10 Department of Physical Education and Sports Sciences, University of Maia (UMAIA), 4475-690 Maia, Portugal

**Keywords:** game performance evaluation tool, decision-making, large-sided games, perception–action, time–motion analysis, sensors

## Abstract

This study investigated the impact of varying the number of small goals on elite female soccer players’ decision-making, technical–tactical skills, running performance, and perceived exertion during game-based situations (GBSs). Sixteen national female players (aged 22.33 ± 2.89 years) participated in three conditions within an 8vs8 game without a goalkeeper (45 × 40 m), each featuring a different number of small goals (1.2 × 0.8 m): (i) 1 small goal (1G); (ii) 2 small goals (2G); and (iii) 3 small goals (3G). Sensors to track players’ positioning, perceived exertion, and notational analysis were used to evaluate player performance. The results indicated that players covered a greater distance at low intensity during the 2G condition compared to both 1G (*p* = 0.024) and 3G (*p* ≤ 0.05). Conversely, the 3G condition promoted a higher distance covered at high intensity compared to 2G (*p* ≤ 0.05). The 1G condition resulted in fewer accelerations (2G, *p* = 0.003; 3G, *p* < 0.001) and decelerations (2G, *p* = 0.012) compared to conditions with additional goals. However, there were no statistically significant effects on technical–tactical actions. Notably, a trend toward improved decision-making was observed in the 1G condition compared to 2G (ES = −0.64 [−1.39; 0.11]) and a longer ball possession duration compared to 3G (ES = −0.28 [−0.71; 0.16]). In conclusion, coaches working with elite female soccer players can strategically vary the number of goals to achieve specific physical aims (i.e., using 2G to emphasize acceleration and deceleration or 3G to promote high-intensity distance) with minimal effects on their perceived fatigue, technical–tactical variables, and decision-making.

## 1. Introduction

In recent years, with the support of FIFA and UEFA, women’s soccer has attracted more participants and contributed to the sport’s growth, spanning from grassroots children’s soccer to elite standards. An evolution in the physical and technical–tactical performance has been observed, contributing to the evolution of the game [1,2].

Notably, the existing literature using global positioning system (GPS) sensors indicates that elite female players typically cover around 10 km during a competitive match at the national team level [1]. Of this total distance, approximately 1.6 km is covered at high speeds (i.e., exceeding 18 km per hour) [2], constituting approximately 16% of the total distance covered. Although this proportion may appear relatively low, high-speed actions have been associated with goal-scoring opportunities [3] and are recognized as key discriminators between performance levels [2]. In fact, elite female players perform 24% to 28% more high-intensity actions compared to players at lower competitive levels [2]. Consequently, developing a comprehensive understanding of how to design appropriate and effective training interventions that enhance both the quantity and quality of high-intensity actions during the match is essential for teams’ performance.

From a technical and tactical perspective, female players typically perform approximately 480 passes per match [4], with an average pass accuracy of 71.5% in Champions League competitions. Their average duel success rate is approximately 51%, with an average of 17 ball losses per match. Further, analysis of elite matches reveals longer possession sequences, fewer long-distance shots, and improved passing and shooting accuracy [5]. An analysis of female players from national teams during friendly tournaments found no significant differences between teams of varying ranking levels (low vs. high) when data was adjusted for playing time [6]. However, previous research related to team success has identified key performance indicators such as goal-scoring opportunities (e.g., scoring first and shots on target relative to the opponent), passing (e.g., assists relative to the opponent), and the percentage of successful duels as critical differentiators between successful and less successful teams [7]. As previously pointed out, such results reinforce the need to develop training interventions that promote the development of players’ technical and tactical skills according to the game requirements.

In line with this approach, coaches often utilize game-based situations (GBSs) during training sessions to enhance players’ tactical, technical, and physical capabilities [8]. GBSs consist of tasks that replicate match-like structures but are conducted in smaller spaces, often with fewer players (small, medium, and large games) and modified rules (conditioned games) compared to official matches [9]. These adaptations aim to improve players’ technical [10] and tactical skills [11], enhance athletic fitness [8], and promote creative decision-making during gameplay [12]. However, the existing literature indicates that different sets of game rules can lead to distinct tactical, technical, and physical responses from players due to variations in the information available or in players’ capacities to perform (i.e., affordances) [13]. Based on this premise, it is essential to understand how different rules can influence women’s performance, enabling coaches to design training environments that effectively support the physical, technical, and tactical development of players according to game demands [13].

Particularly, the existing body of literature on female soccer has explored how the manipulation of space and the number of players can impact players’ performance [14]. For instance, studies have shown that playing on larger pitch sizes leads to more ground covered at high speeds compared to smaller and medium-sized pitches [15]. Additionally, increasing the number of players involved (e.g., 4vs4, 5vs5, 6vs6, 7vs7, 8vs8, and 9vs9) has been found to influence the distance covered at high intensity [16]. When considering technical performance, research has indicated a greater number of successful technical actions in 7vs7 compared to 8vs8 GBSs [17]. Beyond pitch size or player numbers, coaches frequently manipulate the type or number of available targets (e.g., promote defensive compactness or increase time spent in wide channels), which has become an increasingly relevant topic in sports science research.

While most research has explored how different types of targets (i.e., regular goals, small goals, or crossing line) affected youth and adult male players’ physical [18], technical [19], and tactical performance [20,21], fewer studies have considered the impact of the number of targets. From a tactical standpoint, using two small goals instead of one regular goal has been shown to increase team dispersion and player positioning variability, particularly in younger age groups [22]. In professional male players, increasing the number of available targets has afforded more scoring opportunities for the team in possession, leading the defending team to adopt deeper positioning and spend more time covering wide areas [23]. Regarding technical performance, GBSs with three mini-goals and no goalkeeper enabled youth players to receive the ball in more advanced positions with less defensive pressure, resulting in fewer dribbles and more successful outcomes compared to games with a single regular goal [24]. From a physical perspective, playing with fewer targets (e.g., one goal) generally demanded greater physical effort compared to configurations with multiple targets [25]. However, in opposition to the studies developed in male soccer teams, which revealed that changes in the number of goals can have different effects on technical–tactical performance [21], running performance, physiological variables [19], and decision-making [26], such manipulation remains unexplored with female soccer teams [14]. Given the relevance of goal number manipulation in coaching practice, this study aims to investigate the impact of varying the number of goals on the technical–tactical, physical, and physiological parameters among elite female soccer players.

## 2. Materials and Methods

### 2.1. Participants

Sixteen female soccer players from the Lithuanian national team participated in this study (age: 22.3 ± 2.89 years; height: 169.7 ± 5.2 cm; weight: 63.0 ± 6.9 kg; playing experience: 11.6 ± 3.8 years; n = 4 center backs; n = 2 fullbacks; n = 6 center midfielders; n = 2 wingers; n = 2 strikers). All participants trained four to five days per week and competed in weekly matches. Goalkeepers were excluded due to the specificity of their role, which differs substantially from that of outfield players. Prior to participation, each player received an informed consent form and confirmed their voluntary agreement to take part in the study, including consent for the use of anonymized, aggregated data. Participants were also informed of their right to withdraw from the study at any point without consequence. The study protocol was approved by the Ethical Review Board of the Lithuanian University of Sports Sciences Biomedical Research [08.02.2023 MNL-TRS (M)-2023-567].

### 2.2. Study Design

**Figure 1:**
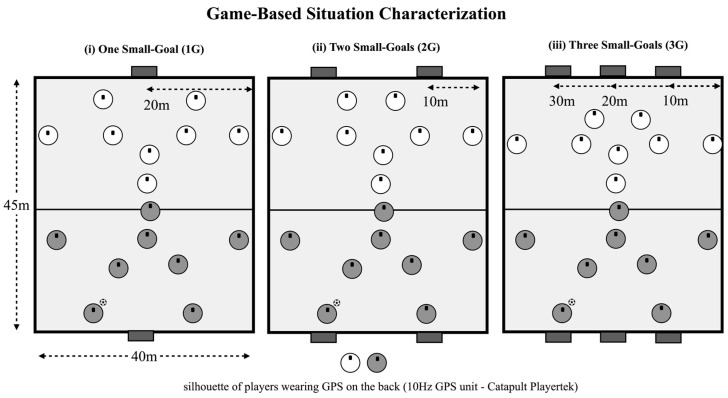
Game-based situations configuration.

The present study utilizes a repeated measures design, where the players were randomly exposed to three experimental conditions (as shown in Figure 1, in which the number of goals was manipulated while keeping constant its size 1.2 × 1.0 m) [23]: (a) 8vs8 ball possession game played with one small goal (1G) positioned in the middle of the endline; (b) 8vs8 ball possession game played with two small goals (2G) placed wide on the pitch; (c) 8vs8 ball possession game played with three small goals (3G). During all conditions, goals were located equally distributed [23]. This game format was selected to involve a higher number of players, thereby promoting more complex tactical behavior. Additionally, having eight players allowed for the implementation of a representative team structure (i.e., defenders, midfielders, and strikers).

### 2.3. Procedures

The study was conducted during the 22/23 season over three non-consecutive testing sessions, which coincided with the preparation camp of the national soccer team. The GBS was embedded within the team’s regular training routine. The first session served as a familiarization period, while the second and third sessions were dedicated to data collection. Before each training session, players engaged in a 20–25 min warm-up that included a 10 min running phase followed by a 5 min mobility-based movements phase. Following the warm-up, players participated in a 5 min technical drills exercise and a 10 min exercise of rondos and positional games (e.g., 4v4 + 3 neutral floaters) to introduce cooperation and opposition interactions before the GBS [27]. Subsequently, the GBS was conducted. All sessions were conducted under consistent environmental conditions, with an average temperature of 8 ± 1 °C, and were scheduled at the same time to minimize potential influences related to circadian rhythms.

For the GBS, the head coach selected the 16 best players and divided them into two balanced teams, considering their tactical understanding, technical skills, physical capacities, and their respective playing roles. The 8vs8 games took place on a 45 × 40 m artificial turf pitch (length × width ratio: 1.125) [28]. Each GBS lasted for 4 min and was followed by a 2 min passive recovery period [10]. To ensure quick restarts when the ball went out of play, numerous balls were strategically positioned around the pitch. No coaching feedback or verbal encouragement was permitted during testing. While the GBS followed official FIFA rules, two exceptions were applied: (i) after a foul or a goal, the ball was promptly put back into play, with coaches passing it from the sidelines to the appropriate team as per the rules; (ii) no offside rule was applied. The attacking team’s objective was to maintain ball possession, progress towards the goal, and score, while the defending team aimed to recover possession of the ball, prevent progression, and avoid the goal. This setup was designed to closely replicate real match scenarios while allowing for the collection of valuable data.

### 2.4. Technical–Tactical Variables and Decision-Making

The games were recorded using a VEO 1 camera (VEO Technologies, Copenhagen, Denmark). The camera was positioned approximately 10 m from the pitch boundaries and elevated to a height of 8 m. Care was taken to ensure that all pitch boundary lines were clearly visible prior to the start of filming. After filming, the footage was processed using the VEO platform with lens correction adjustments and then downloaded to a laptop computer (HP g7 255). Subsequent observational analysis was conducted using Nacsport Scout Plus. The Game Performance Evaluation Tool (GPET), as developed by García-López, González-Víllora [29], was employed to assess players’ decision-making and execution in their technical–tactical actions. This validated tool enables systematic analysis of decision-making and execution quality in four key technical–tactical actions: ball control, passing, dribbling, and shooting [27,29]. Each action was coded as 1 for correct decision-making/successful execution, and 0 for incorrect/unsuccessful actions [29]. Additionally, individual performance indicators were examined, including time under possession (i.e., time spent with the ball during each attacking sequence), number of passes made, shots taken, and dribbles attempted. To assess the team’s technical–tactical actions, the following variables were analyzed: the number of players involved in the attack by touching the ball throughout possession and the team’s ball possession time per attack (i.e., the time from the interception of the ball to its loss).

All video data were analyzed by an experienced soccer match analyst with four years of professional practice. To establish the reliability of the observer, 10% of the total data was re-analyzed by the same observer. A minimum gap of two weeks separated the two rounds of coding. Subsequently, the data obtained from the two observations were compared, and a good reliability rate was determined with an intra-class correlation coefficient (ICC) of 0.83 [30]. This result supports the consistency and trustworthiness of the observer’s assessments.

### 2.5. Locomotor Demands

To assess changes in running performance, 10 HZ GPS sensors (Catapult Playertek, Melbourne, Australia) were used. Each unit (85 mm × 40 mm × 20 mm) was securely positioned in a protective vest located in the upper thoracic area, between the athlete’s shoulder blades. The players always wore the same GPS unit throughout the data collection to ensure interunit reliability [31]. Devices were activated approximately 20 min before the warm-up to ensure that a full and stable satellite signal connection was achieved. The quality of the post-match GPS data was confirmed by examining satellite connectivity and horizontal dilution of precision (HDOP), with average values of 12.3 ± 2.2 satellites and an HDOP of 0.8 ± 0.2, indicating reliable signal reception and positional accuracy. Previous studies have confirmed the validity and reliability of 10 Hz GPS devices for measuring distance and speed during both linear and team sport activities [32]. Five distinct activity profiles were identified based on speed thresholds, as established by Park, Scott [33]. These profiles include walking (0–7 km/h), light running (7–13 km/h), fast running (13–19 km/h), very fast running (19–23 km/h), and sprinting (>23 km/h). In this study, three primary intensity zones were defined to further analyze running performance [34]: a low-intensity zone (<13 km/h), a high-intensity zone (13–19 km/h), and a very high-intensity zone (>19 km/h). The assessment of sprints was excluded due to the constraints of the small pitch configurations used in the study. In addition to speed profiles, the sensors recorded accelerations (counts, >2 m/s^2^) and decelerations (counts, >2 m/s^2^), providing valuable insights into players’ changes in speed and agility [16]. The GPS data were extracted using the Playertek software (Catapult Playertek, Melbourne, Australia) and exported to a Microsoft Excel spreadsheet (Excel, Redmond, WA, USA) for further analysis.

### 2.6. Rate of Perceived Efforts

Participants subjectively rated their rate of perceived effort after the warm-up and immediately after each GBS modification using a custom-designed application on a tablet. A ten-point Borg scale was used [35]. The score of each player was automatically saved on his profile. The subjects were familiar with this scale as it had been used in previous training sessions prior to the study.

### 2.7. Statistical Analysis

All data were assessed for outliers and assumptions of normality using the Shapiro–Wilk test. Descriptive data was presented as mean (M) ± standard deviation (SD). As the data showed both normal and non-normal distributions, the differences between conditions (1G vs. 2G; 1G vs. 3G; 2G vs. 3G) were assessed using parametric tests (Repeated Measures analysis of variance ANOVA) and non-parametric tests (Friedman ANOVA) following previous studies’ approaches that aimed to compare the same players’ performance over different SSG scenarios [10]. Pairwise differences between the conditions were assessed using the Bonferroni post hoc test, while for the non-parametric data, the Durbin–Conover test was used. Additionally, to compare the players’ differences in RPE values from the baseline to post-GBS conditions, the analysis of covariance (ANCOVA) with post-test values as the dependent variable and the pre-test values as the covariate was applied. For each ANCOVA, partial eta-squared (η2) was calculated. Values of 0.01, 0.06, and above 0.14 were considered as small, medium, and large, respectively [36]. Statistical significance was set at *p* < 0.05, and calculations were performed using the Jamovi Project (Computer Software Version 1.2. 2020) [37]. Complementarily, the Cohen’s d effect size (ES, 95% confidence intervals) [36] was calculated for all comparisons using the following thresholds: < 0.2, trivial; 0.2–0.6, small; 0.6–1.2, moderate; 1.2–2.0, large; >2.0 very large [38,39].

## 3. Results

### 3.1. Rate of Perceived Efforts

**Figure 2:**
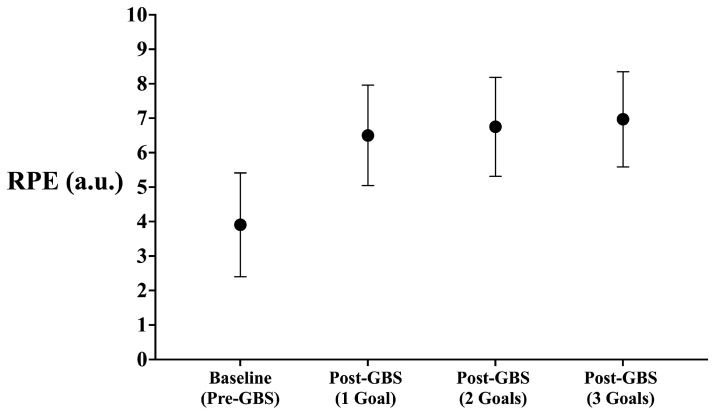
Mean values from the RPE reported by the players at the baseline and the different post-GBS conditions.

Individual and mean changes in players’ RPE after the warm-up and after each GBS are shown in Table 1 and Figure 2. No statistically significant differences were identified between the conditions for the RPE values (F = 0.84, *p* = 0.435). Despite this, the effect size indicated higher values of RPE after the 3G compared to the 1G condition (ES with 95% CI: ES = 0.35 [−0.18; 0.82]).

### 3.2. Locomotor Demands

**Figure 3:**
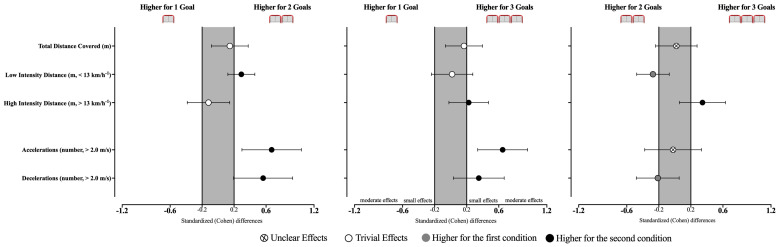
Standardized (Cohen’s d) differences in external load variables according to the different GBS conditions. Error bars indicate uncertainty in the true mean changes with 95% confidence intervals.

The differences between the effects of varying the number of goals in the players’ external load are reported in Table 1 and Figure 3. In general, all GBSs induced similar distances covered (F = 1.19, *p* = 0.312). In contrast, statistically significant effects were identified in the distance covered in low intensity (X^2^ = 4.86, *p* = 0.001), high intensity (F = 3.59, *p* =0.028), and in the number of accelerations (F = 10.0, *p* < 0.001) and decelerations (F = 6.55, *p* = 0.003). Accordingly, players covered a higher distance at the low intensity zone when playing with 2G than when playing with 1G (*p* = 0.024, ES = 0.29 [0.12; 0.46]) or 3G (*p* ≤ 0.05, ES = −0.27 [−0.48; −0.07]). In contrast, playing with 3G led towards higher distance covered at high intensity when compared to 2G (*p* ≤ 0.05, ES = 0.35 [0.06; 0.63]). Additionally, playing with 1G decreased the number of accelerations when compared to 2G (*p* = 0.003, ES = 0.67 [0.30; 1.04]) and 3G (*p* < 0.001, ES = 0.65 [0.34; 0.96]) and the number of decelerations when compared to 2G (*p* = 0.012, ES = 0.56 [0.19; 0.93]).

### 3.3. Game Performance Evaluation Tool (GPET)

**Figure 4:**
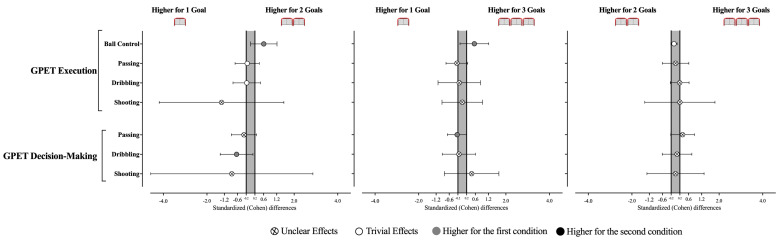
Standardized (Cohen’s d) differences in GPET variables according to the different GBS conditions. Error bars indicate uncertainty in the true mean changes with 95% confidence intervals.

The decision-making and execution (see Table 2 and Figure 4) for the passing, dribbling, shooting, and ball control variables revealed no statistically significant effects were identified between the GBS scenarios (Decision-Making: Passing, X^2^ = 1.20, *p* = 0.550; Dribbling, X^2^ = 2.24, *p* = 0.326; Shooting, X^2^ = 1.27, *p* = 0.529; Execution: Ball Control, X^2^ = 5.20, *p* = 0.074; Passing, X^2^ = 3.67, *p* = 0.159; Dribbling, X^2^ = 2.38, *p* = 0.305; Shooting, X^2^ = 3.45, *p* = 0.178) (Table 2 and Table 3). Despite this, the results derived from the effect size allow some inferences. Accordingly, playing with 1G seems to promote players’ dribbling decision-making when compared to 2G (ES = −0.64 [−1.39; 0.11]). In addition, players demonstrated better ball control execution when playing with 2G and 3G compared to 1G (vs. 2G, ES = 0.61 [0.0; 1.21]; vs. 3G, ES = 0.55 [−0.10; 1.21]).

### 3.4. Individual and Team Technical–Tactical Variables

**Figure 5:**
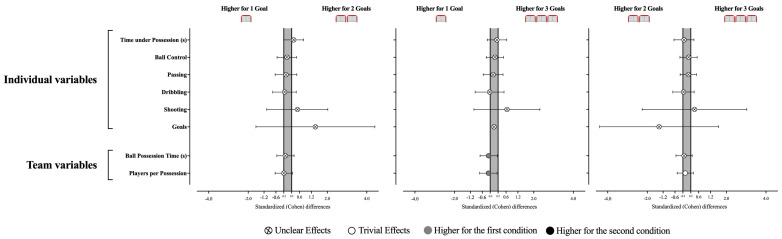
Standardized (Cohen’s d) differences in individual and tactical variables according to the different GBS conditions. Error bars indicate uncertainty in the true mean changes with 95% confidence intervals.

No statistically significant differences were found between the GBS modifications in terms of individual (see Table 3 and Figure 5; Time under possession, X^2^ = 0.96, *p* = 0.618; Ball control, X^2^ = 0.788, *p* = 0.674; Passing, X^2^ = 0.602, *p* = 0.740; Dribbling, X^2^ = 0.545, *p* = 0.761; Shooting, X^2^ = 0.667, *p* = 0.717; Goals, X^2^ = 4.00, *p* = 0.135) and team technical–tactical variables (Ball Possession Time, X^2^ = 0.958, *p* = 0.619; Players per possession, X^2^ = 0.712, *p* = 0.700). Despite this, the team tends to spend more time in possession during the 1G than in 3G (ES = −0.28 [−0.71; 0.16]), which may result from involving more players in 1G than in 3G (ES = −0.29 [−0.73; 0.15]).

## 4. Discussion

The aim of this study was to determine how the number of goals and their orientation/location in the GBS influence the decision-making, technical–tactical actions, physiological, and running performance of elite female soccer players. The main findings suggest that changes in the number of goals affect the distance covered by the players in both low- and high-intensity zones, and the number of accelerations and decelerations (*p* < 0.05). However, only some effects were observed in terms of perceived fatigue, technical–tactical variables, and decision-making, without statistically significant changes (*p* > 0.05).

### 4.1. Physical Variables

Examining the characteristics of a soccer match, it can be observed that high-performance female soccer players cover an average of 108 ± 10 m/min [40] and perform slightly over 400 accelerations and decelerations [16]. Our study yielded similar results, as the distance covered by female soccer players during different GBS modifications ranged from 111.93 ± 11.30 m/min to 113.84 ± 12.07 m/min. However, due to the dynamic and volatile nature of soccer, the distance covered by players at a given speed varies. The results revealed that most of the distance was covered at low intensities. The reason for such values may be linked to the limited playing space in relation to the number of players in the GBS. Previous research has indicated an increase in the distance covered at high speed when the available space to play is increased [9]. Moreover, it is important to note that recent research involving top-class female players during UEFA international competitions revealed that match demands are highly intermittent, with brief passages of extremely high intensity interspersed throughout the game [41]. This suggests that when designing training tasks, coaches should replicate these most demanding passages to adequately prepare elite players. In accordance, it is plausible to assume that the restricted space in the current GBS formats constrained opportunities for high-intensity movements, resulting in lower distances covered at higher speeds.

When comparing the physical variables across the experimental conditions, the results revealed a higher number of accelerations and decelerations when playing with 2G and 3G. These findings were somewhat anticipated since increasing the number of goals per team from 1 (i.e., a regular goal) to 3 (small goals) led players to spend more time in the defensive sector and wide areas, and to perform a higher number of changes in direction to protect the shooting lines according to changes in ball position, as indicated in previous research [23]. As a result, it is likely that the defensive team may adopt a more conservative positioning due to the increased scoring opportunities, while the offensive team aims to move the ball to create space for progression. Consequently, this dynamic coadaptation between the offensive and defensive teams may contribute to a higher number of adaptive movements to cover the space and, consequently, a higher number of accelerations and decelerations. Indeed, previous studies have shown that accelerations are more prevalent during dribbling and change-of-direction actions [15], which are likely to emerge within the confrontation interactions developing between teams. These accelerations result from the continuous individual and collective positional adjustments made by the defenders and attackers to protect the goals or, in opposition, to create space against a compact defensive line. Hence, coaches may consider incorporating additional training targets to emphasize players’ acceleration and deceleration profiles. However, the distinction between 2G and 3G may depend on the specific targeting strategies employed by coaches and technical staff. For instance, playing with 3G led to an increase in the distance covered in the high-intensity zone (i.e., Z2). This suggests that players may have attempted to elevate the tempo of the game by passing the ball at a higher rate, as indicated by the high mean values for the passing variable and the lower mean values for ball possession time. This shift in strategy may be aimed at destabilizing the opposition while maintaining defensive vigilance. Consequently, the 3G condition may be more suitable during a short-term regular microcycle (i.e., a cycle with four training sessions between games), where the MD−3 (match day minus 3) session structure emphasizes both acceleration/deceleration profiles and encourages higher distance covered, as previously suggested [42]. In fact, recent research analyzing four consecutive microcycles in professional female players from the Spanish first division reported that MD−3 consistently registered the highest external loads across all variables, including total distance, high-intensity running, sprint distance, and number of accelerations and decelerations [43]. These findings emphasize the strategic role of MD-3 in eliciting peak physical loads.

### 4.2. Rate of Perceived Efforts

There was no statistically significant difference in terms of perceived fatigue (*p* > 0.05). Similar results have been reported in other studies. For instance, the 1G and 2G modifications, as well as the line rule (stopping the ball after crossing the line), did not lead to statistically significant changes in the RPE (Rate of Perceived Exertion) scores of young soccer players [44]. A comparable trend in RPE ratings was observed among university students when the 1G and 2G modifications were applied [45]. Consequently, the findings suggest that varying the number of goals does not result in a significant difference in terms of perceived fatigue [44,45]. This lack of difference may be attributed to the fact that, across all experimental conditions, the relative pitch area per player and the number of players involved remained constant. However, it is important to recognize that RPE is not solely influenced by the external workload but is also shaped by internal and contextual factors, such as psychological stress, sleep quality, and recovery status [46], which were not considered in this study and may have impacted the results. Still, parameters are widely recognized as key determinants of both internal and external load [47]. Therefore, coaches can adjust the number of goals to create distinct physical stimuli (e.g., using 2G to emphasize acceleration and deceleration, or 3G to prioritize distance covered) without significantly affecting players’ internal perception of effort and the physiological response to the exercise.

### 4.3. Decision-Making and Technical–Tactical Indicators

In modern soccer, increasing attention is being devoted to the tactical and technical training of players, with a primary focus on improving decision-making skills [12,27]. Accordingly, one of the most effective ways to enhance decision-making and the performance of technical–tactical actions in soccer is the use and manipulation of GBSs (Goal-Based Scenarios) [12]. Depending on the primary training objective, coaches often design GBSs without official goals to emphasize players’ physical development [48]. However, as previously noted, modifying the rules or structure of the game not only influences physical demands but also impacts players’ spatial positioning and technical–tactical behavior [11]. Although it might have been expected that differences in players’ decision-making and execution of technical–tactical actions would emerge, no statistically significant differences were observed in the present study. This may be partly attributed to the relatively small sample size and the fact that the analysis did not categorize actions according to game principles [49,50]. In addition, it may also be possible that the location of the goals (i.e., positioned in the wide channels or central channel) may also contribute to the lack of results. That is, positioning goals in the wide channels tends to encourage more lateral passes and directional changes, while placing goals in central areas may stimulate more forward and penetrative passing [51]. It is also important to note that most studies exploring the differences in the number of goals adopted 5-a-side, 7-a-side, or 11-a-side targets [22,23], which may have contributed to more pronounced tactical differences between conditions. In contrast, the present study modified the number of small goals, while keeping its size constant. That is, it is accepted that teams face more difficulties in protecting 2G when using regular-sized goals [22,52], as a result of the higher exploration of wide channels by the offensive team [23,52], which may result in more opportunities to shoot. While the same strategy may be adopted by teams when increasing the number of small goals (i.e., switch play), proper positioning and defensive movement (which can be depicted by accelerations and decelerations) may allow teams to limit the chances to score, similar to 1G. Altogether, it may have contributed to the lack of statistical differences in players’ decision-making and execution, and consequently, it would be interesting if further research considers exploring the cumulative effect of adding more goals in relation to the location.

Nevertheless, some trends can be identified. Notably, playing with 1G led to a longer time in possession with a lower external load. These findings are aligned with a previous study comparing the technical–tactical effects of playing with 1G vs. 2G, revealing a higher game volume with fewer goals [53]. Accordingly, it is expected that the defensive team would adopt a compact defensive behavior in front of the goal [23], and consequently, the team under possession may have to take more risks (e.g., engaging in dribbling). In fact, the literature has shown a higher number of inaccurate passes, ball losses, and recoveries when playing with 1G than 2G, which may reflect this intention of assuming more risks to overcome the defensive strategy adopted by the team without possession [19,21]. In contrast, previous research has found that increasing the number of goals increases the number of goal-scoring opportunities [23], aligning with the higher number of goals scored in the 3G condition. These results seem to support the perspective that scoring against 1G may compel players to explore adaptive behaviors to disrupt the opposing team. For instance, players may have adopted one of two different strategies [21,54]: (i) utilizing passing to manipulate defenders, as evidenced by longer ball possession duration, a greater number of passes, and more players involved per possession; or (ii) employing dribbling to generate additional space for progression. Our results differ from those of Fenoglio [52], who found a greater number of dribbles under the 2G condition in prepubescent players; such discrepancies may stem from differences in participants’ age and skill level. Importantly, our study involved elite female players with fully developed technical repertoires and included a qualitative analysis of actions, assessing not only the frequency but also the decision-making and technical execution. This provides a more comprehensive understanding of performance compared to Fenoglio’s study [52], which focused solely on the number of actions. Based on our results, coaches may consider using the 1G condition to stimulate dribbling with tactical intent, while increasing the number of goals could be useful to encourage a higher frequency of offensive actions such as shooting.

To the best of our knowledge, this study represents the first of its kind to investigate the impact of the number of goals on decision-making, technical–tactical actions, physiological, and running performance among national-level female soccer players. Coaches often use GBSs with different numbers of goals during earlier phases of the training session to develop the team’s tactical principles and patterns, and therefore, the results from this study may help in better planning and scheduling the manipulation of the number of goals. While the findings offer valuable practical insights, it is important to note some limitations. Firstly, it is important to acknowledge that the findings of this study are specific to the GPS technology used (Catapult Playertek, Melbourne, Australia). Previous research has shown that different GPS brands may produce varying outputs, particularly regarding acceleration and deceleration metrics [32]. Therefore, caution is advised when comparing these results with studies using alternative tracking systems, as inter-device variability may influence the measured performance indices. Further, the study could be enhanced by including a more detailed tactical analysis, particularly by separating data according to game phases (offensive vs. defensive) and considering the behavior of players without the ball (e.g., supporting players). Moreover, the use of player positioning data obtained from alternative systems (e.g., automatic video tracking systems) combined with advanced video features could enable the integration of physical, tactical, and technical variables. This multi-dimensional approach would provide a more comprehensive and ecologically valid view of player behavior in game-based scenarios. Further, although the sample consisted of elite-level players, the relatively small group size may constrain the broader applicability of the results and reduce sensitivity in capturing more subtle differences in performance. Future research should aim to involve larger samples to strengthen statistical power and generalizability, while also contrasting players from different performance levels (e.g., national vs. club-level) and age groups (e.g., adult vs. youth), as previous studies have indicated that female players may exhibit varying performances based on the specific context [1]. Such comparisons could provide a more nuanced understanding of how goal configurations affect player behavior across varying competitive contexts. In addition, including tactical variables (e.g., team synchrony) and the decision-making level of offensive players without the ball (i.e., supporting players) would contribute to a more holistic understanding of players’ performance.

## 5. Conclusions

In general, varying the number of goals during an 8vs8 GBS with national-level female players produced notable effects on running performance variables. Specifically, playing with 2G emphasized acceleration and deceleration, whereas playing with 3G resulted in a higher distance covered at high intensity. This suggests that the inclusion of additional goals encourages the defensive team to adopt a closer defensive stance, prompting the offensive team to maneuver the ball to create space. Consequently, it places greater emphasis on shorter, high-intensity actions by the defensive team to press the player in possession and maintain tighter passing lines. Alternatively, in scenarios with more goals (i.e., 3G), the offensive team may be compelled to play at a faster pace, despite the increased scoring opportunities due to defensive compactness. Concerning technical–tactical actions, the results did not exhibit statistically significant effects, which may be related to the analysis focused on the player under possession without considering the contextual principles (i.e., maintaining possession or progression) or the movements of the teammates without the ball. However, a trend toward improved decision-making during dribbling was observed in the 1G condition. These findings might be attributed to the shorter distance between teams, necessitating a more sophisticated offensive approach or the use of dribbling to create space.

In summary, coaches can strategically adjust the number of goals during GBSs to modify task emphasis, targeting different physical stimuli without significantly impacting players’ perceived effort. Specifically, 2G and 3G can be employed to enhance acceleration and deceleration actions, while 3G can also promote increased distance covered at high speed. Regarding technical–tactical actions, 1G may be suitable to encourage players’ dribbling decision-making, while 2G and especially 3G can be utilized to increase the frequency of shooting actions.

## Figures and Tables

**Table 1:** Descriptive statistics (Mean ± SD; Raw ± 95% CI) and inferential statistics from the internal and external load according to the game scenarios.

Variables	1 Goal	2 Goals	3 Goals	Difference in Means (Raw ± 95% CI)			*p*
	Mean ± SD	Mean ± SD	Mean ± SD	1G vs. 2G	1G vs. 3G	2G vs. 3G	
Rate of Perceived Exertion (RPE)							
Post-GBS (a.u.)	6.5 ± 1.5	6.8 ± 1.5	7.0 ± 1.4	0.3; ±0.8	0.5; ±0.7	0.2; ±0.7	0.435
External Load							
TD (m/min)	111.93 ± 11.30	113.62 ± 10.05	113.84 ± 12.07	6.79; ±10.63	7.66; ±10.67	0.87; ±11.95	0.312
TD in low-intensity zone (m/min)	95.34 ± 8.03	97.9 ± 8.22	95.49 ± 9.23	2.56; ±1.49	0.16; ±2.27	−2.4; ±79	**0.001 ^a,c^**
TD in high-intensity zone (m/min)	15.03 ± 7.96	14.46 ± 5.93	17.10 ± 7.33	−0.97; ±13	1.81; ±1.98	2.78; ±2.31	**0.028 ^c^**
TD in very high-intensity zone (m/min)	1.56 ± 2.22	1.26 ± 1.76	1.25 ± 1.39	2.88; ±1.6	2.78; ±1.34	−0.09; ±1.52	0.402
Accelerations (counts; >2 m/s)	14.5 ± 4.27	17.38 ± 4.14	17.28 ± 4.03	2.81; ±1.85	1.75; ±1.58	−1.06; ±1.33	**<0.001 ^a,b^**
Deaccelerations (counts; <−2 m/s)	16.56 ± 5.35	19.38 ± 4.71	18.31 ± 4.37	6.79; ±10.63	7.66; ±10.67	0.87; ±11.95	**0.003 ^a^**

Note: m = meters; TD = total distance covered; n = number. The bold values mean significant differences. Letters represent differences according to the following conditions: (a) 1G direction vs. 2G; (b) 1G direction vs. 3G; (c) 2G vs. 3G.

**Table 2:** Descriptive statistics (Mean ± SD; Raw ± 95% CI) and inferential statistics from the Game Performance Evaluation Tool (GPET) according to GBS.

Variables	1G	2G	3G	Difference in Means (Raw ± 95% CI)			*p*
	(Mean ± SD)	(Mean ± SD)	(Mean ± SD)	1G vs. 2G	1G vs. 3G	2G vs. 3G	
Decision Making							
Passing	0.83 ± 0.25	0.69 ± 0.35	0.76 ± 0.24	−0.09; ±0.17	−0.07; ±0.13	0.10; ±0.16	0.550
Dribbling	0.87 ± 0.26	0.76 ± 0.33	0.88 ± 0.30	−0.20; ±0.24	−0.05; ±0.24	0.06; ±0.14	0.326
Shooting	0.50 ± 0.50	0.40 ± 0.55	0.36 ± 0.46	−0.50; ±2.15	0.25; ±0.72	0.00; ±0.76	0.529
Technical-Tactical Skill Execution							
Ball Control	0.96 ± 0.09	1.0 ± 0.0	0.99 ± 0.02	0.04; ±0.04	0.03; ±0.04	0.00; ±0.01	0.074
Passing	0.84 ± 0.24	0.75 ± 0.35	0.75 ± 0.27	−0.05; ±0.17	−0.07; ±0.15	0.00; ±0.18	0.159
Dribbling	0.90 ± 0.27	0.83 ±0.29	0.84 ± 0.36	−0.06; ±0.21	−0.05; ±0.32	0.02; ±0.22	0.305
Shooting	0.28 ± 0.44	0.30 ± 0.44	0.32 ± 0.42	−0.67; ±1.43	0.00; ±0.47	0.10; ±0.81	0.178

**Table 3:** Descriptive statistics (Mean ± SD; Raw ± 95% CI) and inferential statistics from the individual and team technical–tactical variables across GBSs.

*Variables*	1G	2G	3G	Difference in Means (Raw ± 95% CI)			*p*
	(Mean ± SD)	(Mean ± SD)	(Mean ± SD)	1G vs. 2G	1G vs. 3G	2G vs. 3G	
*Decision-Making*							
Passing	0.83 ± 0.25	0.69 ± 0.35	0.76 ± 0.24	−0.09; ±0.17	−0.07; ±0.13	0.10; ±0.16	0.550
Dribbling	0.87 ± 0.26	0.76 ± 0.33	0.88 ± 0.30	−0.20; ±0.24	−0.05; ±0.24	0.06; ±0.14	0.326
Shooting	0.50 ± 0.50	0.40 ± 0.55	0.36 ± 0.46	−0.50; ±2.15	0.25; ±0.72	0.00; ±0.76	0.529
*Technical–Tactical Skill Execution*							
Ball Control	0.96 ± 0.09	1.0 ± 0.0	0.99 ± 0.02	0.04; ±0.04	0.03; ±0.04	0.00; ±0.01	0.074
Passing	0.84 ± 0.24	0.75 ± 0.35	0.75 ± 0.27	−0.05; ±0.17	−0.07; ±0.15	0.00; ±0.18	0.159
Dribbling	0.90 ± 0.27	0.83 ±0.29	0.84 ± 0.36	−0.06; ±0.21	−0.05; ±0.32	0.02; ±0.22	0.305
Shooting	0.28 ± 0.44	0.30 ± 0.44	0.32 ± 0.42	−0.67; ±1.43	0.00; ±0.47	0.10; ±0.81	0.178

## Data Availability

In order to protect the subjects’ confidentiality and privacy, data are only available upon request. Interested researchers may contact the board from the Research Center in Sports Sciences, Health Sciences, and Human Development to request access to the data (cidesd.geral@utad.pt).
